# Annotations

**Published:** 1908-11-07

**Authors:** 


### Annotations.

### The Research Defence Society.

Mr. Stephen Paget's address on the Work of the Research Defence Society, recently delivered before the North London Medical and Chirurgical Society, and published in the last number of the *British Medical Journal*, deserves to be read and studied by members of every branch of the medical profession. It is indeed unfortunate that those who are continually putting to practical uses the work of the pioneers of scientific research should have so little time or opportunity for enlightening the public on the true facts of experiments upon animals, and thus combating the unscrupulous attacks of the anti-vivisectionists. But, however busy they may be, medical practitioners can at least arm themselves with some of the essential arguments in favour of experimental research, so that, when their opinion is asked by those who are in doubt, they may have a cogent word to speak in season. The members of our profession are notoriously shy of platforms and public controversies, even when these are concerned with matters of vital importance to themselves and their patients; but there is always the danger that diffidence may be mistaken for apathy. It is evident from the columns of the London and provincial journals that the anti-vivisectionist officials, perhaps in consequence of the evidence given before the last Royal Commission and its damaging effect upon their cause, have lately shown signs of feverish activity. The general public has happily not yet lost its common sense, its sense of humour, or its sense of proportion. But even the grossest of lies, if it is repeated frequently enough, and not denounced as often as it appears, begins at last to assume an air of truth; and the anti-vivisectionists, who are swept along for the most part by good motives diverted into bad channels, deal especially in "the lie which is half a truth," which, as is well known, is "ever the blackest of lies." The medical profession owes it to itself and to those for whom it works to stem the torrent of inaccuracies, insinuations, and abuse, which still pours out from the offices of the fourteen or fifteen rival anti-vivisection societies. Single-handed effort can do something, indeed it can do much; but combination and organisation can do more. We have on several previous occasions drawn attention to the objects and methods of the Research Defence Society, and we would again urge those of our readers who have not already put themselves in touch with it to do so now, before other affairs thrust the matter out of their minds. Whatever Mr. Paget writes is certain to give pleasure in the reading, and his recent paper not only conveys information which every medical man should possess, but has also a literary quality which in itself would justify a sympathetic perusal. It is, of course, far from the aim of the Research Defence Society to appeal to medical men alone; but it is essential for the fulfilment of the Society's objects that its indefatigable honorary secretary and the many distinguished representatives of Church and State, and of the non-medical arts and sciences who hold office or membership in it, should have not only the moral support and encouragement, but also the active co-operation of the rank and file of the profession. The Society has already accomplished much good work, but there remains a great deal still to be done, and help is needed.

### Is our Climate Changing?

Accustomed to the vagaries of a climate which must surely be the most inconstant in the world, even the stolidity of the average Briton has been a little disturbed by the summer-like heat of the month of October just passed. This mildness of the weather, which has provoked already many statistical tables in the daily Press, lends additional point and interest to the paper bearing the above title, read in the previous month before the British Association by Sir John Moore. This is a very general impression abroad, as he points out, that winters are less severe and later than of old, that summers are rainier and cooler, springs less balmy, and autumns more torrid than in the good old times. Certainly it is common experience that snow rarely falls in any quantity before January, whereas all old traditions represent a heavy snowfall as the proper environment for Christmas festivities. Probably, however, the inclemency of December and the joyousness of April are the outcome of poetic license rather than of the actual experience of our forefathers. So at least concludes Sir John Moore, and he supports his thesis with some very remarkable evidence. Perhaps the most interesting, undoubtedly the most venerable, of the documents quoted is the weather journal of the Rev. William Merle, a Lincolnshire rector, for the years 1337-1343. Thus in 1341 he records weather in April which must have caused as much disgust among farmers as it so often does now. From the 6th to the 13th of the month there was continued frost, diversified at frequent intervals by snow and hail. In the following year "there was spring-like weather the whole time between September and the end of December," so much so that leeks and cabbages blossomed in that season. This MS. journal is in the Bodleian Library at Oxford, and it certainly affords good proof that neither in respect of variability nor of the date of the seasons has our climate changed appreciably within six hundred years. There is, unfortunately, no information as to death rates in the remote days of Plantagenets, otherwise instructive comparisons of the results of particular climatic conditions then and now might be possible. Thus January, 1341, was of a kind to test the resistance of the toughest Englishman. We read that hard frosts, bright warm days, heavy rains, high winds, and so on, succeeded one another with the kaleidoscopic rapidity we know so well in this twentieth century. Sir John Moore has accumulated an enormous amount of data, on which his main conclusion is based, and this the curious will find in the October issue of the *Dublin Journal of Medical Science*. His other important deduction is that no trace of cyclic seasonal variation is traceable in these islands during the last 130 years.

